# A single dose of quadrivalent human papillomavirus (HPV) vaccine is immunogenic and reduces HPV detection rates in young women in Mongolia, six years after vaccination

**DOI:** 10.1016/j.vaccine.2020.04.041

**Published:** 2020-06-02

**Authors:** Tsetsegsaihan Batmunkh, Marguerite T. Dalmau, Margad-Erdene Munkhsaikhan, Tungalagtuya Khorolsuren, Narantuya Namjil, Unursaikhan Surenjav, Zheng Quan Toh, Paul V. Licciardi, Fiona M. Russell, Suzanne M. Garland, Kim Mulholland, Claire von Mollendorf

**Affiliations:** a National Cancer Council of Mongolia, #101, Oyutnii khotkhon 68/1, Bayanzurkh district, Ulaanbaatar, Mongolia; b Onoshmed Laboratory, Khatagtai Hospital, Sukhbaatar District, Ulaanbaatar, Mongolia; c National Center for Public Health, Olympic Street 2, Ulaanbaatar, Mongolia; d Murdoch Children’s Research Institute, 50 Flemington Road, Parkville, Melbourne, Australia; e Centre for Women’s Infectious Diseases Research, Royal Women’s Hospital, Parkville, Melbourne, Australia; f Department of Obstetrics and Gynaecology, University of Melbourne, Parkville, Melbourne, Australia; g Department of Paediatrics, The University of Melbourne, Parkville, Melbourne, Australia; h Department of Infectious Disease Epidemiology, London School of Hygiene and Tropical Medicine, London, UK

**Keywords:** Human papillomavirus, Single dose, 4vHPV, HPV vaccine schedule

## Abstract

**Background:**

Emerging observational evidence suggests a single-dose of human papillomavirus (HPV) vaccine may be protective against vaccine-targeted HPV infection and associated cervical dysplasia. We aimed to demonstrate whether a single dose of quadrivalent HPV (4vHPV) vaccine was immunogenic and reduced HPV detection rates in young women in Mongolia. We also assessed knowledge and attitudes regarding HPV and the HPV vaccine.

**Methods:**

A retrospective paired cohort study was undertaken to evaluate the effect of a single dose of 4vHPV, given at age 11–17 years in 2012, on HPV detection rates, when compared with unvaccinated women. Real time PCR was performed on self-administered vaginal swabs for HPV detection. An immunological analysis detecting neutralising antibodies (NAb) to high-risk HPV (HRHPV) genotypes 16 and 18 was performed on sera from a subset of 58 participants. Questionnaires evaluated knowledge, attitudes and self-swab acceptability.

**Findings:**

A total of 475 women (mean age 20.4 years ± 1.6) were recruited; 118 vaccinated and 357 unvaccinated women. The prevalence of vaccine-targeted HRHPV16 and 18 was reduced by 92% (95%CI 44–99%) in the vaccinated (1·1%) compared with the unvaccinated (15.4%) group. The percentage of non-vaccine HPV genotypes was similar between vaccinated (26.5%) and unvaccinated (26.7%) groups. Approximately 90% and 58% of vaccinated women remained seropositive after six years for HRHPV16 and 18, respectively, with neutralising antibody levels 5- and 2-fold higher than unvaccinated women (p < 0.001).

**Interpretation:**

One dose of 4vHPV vaccine reduces vaccine-targeted HPV genotypes, six years following vaccination, with high levels of HR genotype seropositivity among young Mongolian women.

## 1 Introduction

Since initial licensure in 2006, the three internationally licensed HPV vaccines (bivalent [2vHPV], quadrivalent [4vHPV] and nonavalent [9vHPV]) have been demonstrated as highly efficacious in protecting against vaccine-type HPV infection [1], [2]. In 2014, the World Health Organization (WHO) amended its scheduling recommendation [3]. Originally administered as a three-dose schedule, the 2vHPV and 4vHPV were amended to a two-dose schedule separated by six months for individuals under 15 years of age based on non-inferior immunogenicity studies in girls who received two doses and women (15–26 years old) who received three doses. Since then, emerging evidence from observational studies suggest that one dose of vaccine is protective against HPV infection and associated diseases [4], [5], [6], [7], [8].

The disparity in cervical cancer burden is evident with 90% of cervical cancer-related deaths occurring in low and middle-income countries (LMICs) [9]. Resource constraints pose a significant barrier to increased capacity for prevention, early diagnosis, screening and treatment in these settings. Although since 2016, Gavi, the Vaccine Alliance, has allowed countries to commence national vaccine programs directly, without prior demonstration projects [10], the necessary financial commitment and logistics remain a major barrier for LMICs [11]. Demonstrating long-term efficacy of a single dose of the HPV vaccine has the potential to combat these barriers.

Observational data on the efficacy of one dose of 2vHPV and 4vHPV have emerged as a result of interrupted and thus incomplete dosage regimens in randomised controlled trials or demonstration campaigns. Combined analyses of both the Costa Rica Vaccine Trial and PATRICIA Trial reported that one dose of 2vHPV could be as efficacious as three doses when using the endpoint of persistent HPV infection accumulated over the four-year trial period [4], [5]. This is also supported by antibody data, where antibody levels were nine times higher than those from natural infection and persisted out to at least seven years. [4]. The suspension of a multi-centre cluster randomised trial in India in 2009 resulted in 4950 participants receiving only one dose from a three-dose regimen of 4vHPV. Observational data from this group indicated a robust immune response against HRHPV 16 and 18 that remained stable over a four-year period [6]. Small-scale observational studies succeeding vaccine demonstration projects of the 2vHPV in Uganda [7] and 4vHPV in Fiji [8] also demonstrated persisting immunogenicity from one dose of vaccine. While the antibody levels remained inferior to higher dose recipients, one-dose participants from both studies elicited higher antibody levels than unvaccinated women towards vaccine-targeted HPV types [7], [8].

The Axios Foundation, through the Millennium Challenge Account, donated 44,800 4vHPV doses to the Mongolian Ministry of Health in 2012 [12]. Due to community resistance, the campaign did not vaccinate the intended 14,063 girls with a three-dose regimen. A total of 9111 girls aged 11–17 years were vaccinated and coverage was 64.9%, 75.4% and 77.4% for three, two and one dose, respectively [12]. Reduced doses were primarily due to parent withdrawal. In 2018, we explored the effect of the 2012 campaign on reducing vaccine-targeted HPV infection among three-dose recipients in Mongolia [13]. The current study extends these findings to one-dose recipients to assist country-level vaccine introduction and contribute to the global evidence for one-dose HPV schedules. The aim of our study was to identify whether vaccination with a single dose of 4vHPV, six years prior, was immunogenic and associated with reduced HPV detection rates in young women aged 16–26 years in Mongolia when compared to an unvaccinated group. A secondary aim was to explore knowledge and attitudes regarding HPV, the vaccine and cervical cancer as well as self-swab acceptability in the same groups.

## 2 Methods

### 2.1 Study design and participants

This retrospective paired cohort study was conducted between September 2018 and February 2019. Women were recruited from the Bayangol and Baganuur districts of Ulaanbaatar city and the Umnugobi and Selenge provinces. Unvaccinated participants were also recruited from the Sukhbaatar district of Ulaanbaatar city and Arkhangai province; neighbouring and socioeconomically similar sites. Women who received one dose of 4vHPV in the 2012 pilot vaccination campaign were frequency age-matched to a group of women who never received any HPV vaccine from the appropriate district or province.

Recruitment methods were similar to those employed in our previous study [13]. Eligible vaccine recipients were identified through immunisation records at the National Center for Communicable Diseases, Ulaanbaatar. An attempt was made to contact each one-dose recipient for invitation to the study. Peer-referral was the preferred method of recruitment for the unvaccinated group on the assumption it would increase comparability between the two groups for socioeconomic and behavioural factors. When peer-referral was insufficient to reach target numbers, participating health centres and student dormitories were approached. All participants were de-identified by unique participant IDs according to study site. A participant information sheet and consent form were provided in Mongolian language and individual written informed consent was received by all participants.

All study participants were asked to complete a questionnaire which included demographic characteristics, sexual and reproductive history for information on possible confounders, as well as knowledge and attitudes towards HPV, HPV vaccine and cervical cancer.

Participants were requested to collect a self-administered vaginal swab for HPV detection and to complete a short questionnaire regarding the acceptability of this process. Blood samples for serological analysis were sequentially collected from a subset of consenting vaccinated and unvaccinated women within the Bayangol district of Ulaanbaatar. Blood collection was only performed in the capital city to limit time to processing and ensure sample consistency.

### 2.2 Questionnaire methods

The first questionnaire utilised semi open-ended questions to collect participant demographics including sexual and reproductive history and relevant lifestyle risk factors. Combined open and closed-ended questions were used to determine participant knowledge and attitudes on HPV, the HPV vaccine and cervical cancer, which was scored based on correct responses. The questionnaire design was based on our larger HPV genoprevalence study conducted between 2017 and 2018 with recipients who received three doses of 4vHPV within the same sites [13]. The second questionnaire on self-test acceptability used closed-ended questions and an additional section for open feedback to determine ease of use, associated pain or discomfort and perceived benefits of the self-swab method. Questionnaires were delivered to participants via paper-based forms.

### 2.3 Laboratory methods

Self-administered vaginal swabs (Copan, Italy) were requested from all study participants. The laboratory methods for HPV detection (Supplementary file 1) were consistent with the methods used in our previous study of three-dose 4vHPV recipients in Mongolia [13]. It is important to note that the Xpert HPV Assay (Cepheid Inc, Sunnyvale, CA, USA) identifies HRHPV16 and HRHPV18/45 types in two distinct detection channels, and reports 11 other high risk types (P3 channel: 31, 33, 35, 52, 58; P4 channel: 51, 59; P5 channel: 39, 56, 66 and 68) in a pooled result. Samples testing positive for HRHPV 18/45 were retested using the Anyplex II HPV 28 (Seegene, Korea) which can distinguish 28 HPV genotypes (6, 11, 16, 18, 26, 31, 33, 35, 39, 40, 42, 43, 44, 45, 51, 52, 53, 54, 56, 58, 59, 61, 66, 68, 69, 70, 73, 82).

For the immunological analysis, 5 mL of blood was collected in a serum gel tube from a subset of participants enrolled in Ulaanbaatar (n = 58; 30 vaccinated and 28 unvaccinated). The blood was processed at Onoshmed laboratory, Ulaanbaatar. Serum samples were stored at −80 °C and shipped on dry ice to the Murdoch Children’s Research Institute, Australia for testing. An HPV pseudovirion-based neutralisation assay was used to detect neutralising antibodies (NAb) to HRHPV16 and 18 [7]. The effective dose 50 (ED50) is the highest serum dilution that reduces the secreted alkaline phosphatase activity by at least 50% in comparison to the control (pseudovirions without serum). A sample with an ED50 value of ≥ 100 was considered HPV seropositive; seronegative samples were given a value of 50.

### 2.4 Study outcomes

The primary outcome was defined as detection of HPV high-risk genotype 16 and/or 18 in the vaccinated and unvaccinated groups. Secondary outcomes included; detection of other HRHPV genotypes in the same groups; neutralising antibody titers to HRHPV16 and 18 six years following 4vHPV among a subset of women from each group; knowledge and attitudes of young Mongolian women in relation to HPV, the vaccine and cervical cancer; and the acceptability of self-administered vaginal swabs based on open and closed ended questions.

### 2.5 Statistical analysis

A maximum of 240 one-dose vaccinated women were potentially eligible for enrolment based on immunisation records from the 2012 pilot vaccination campaign. From available HPV prevalence data in Mongolia [14], it was estimated that HRHPV16 and 18 would be detected in 18% of unvaccinated women aged 16–26 years. After significant recruitment efforts (Supplementary file 2), a total of 118 women vaccinated with one dose of 4vHPV were recruited into the study. To increase study power to detect a difference between the vaccinated and unvaccinated groups, we recruited 357 unvaccinated participants (3:1 ratio). The recruited numbers had > 90% power to demonstrate a difference in HPV prevalence between the groups at p < 0.05 level.

Categorical demographic variables were summarised as numbers and percentages and continuous variables using means and standard deviations for the vaccinated and unvaccinated groups. Chi squared tests were used to compare differences between the groups. HPV prevalence was assessed by the following genotype sub-groups; all tested high-risk types, vaccine-targeted types 16 and 18 (both together and separately), high-risk types excluding HRHPV16 and 18 and co-detection with multiple genotypes. Prevalence ratios were calculated by comparing the prevalence between vaccinated and unvaccinated groups. Within each HPV sub-group, univariable log-binomial regression models were fitted to estimate unadjusted prevalence ratios of HPV positivity in association with participant characteristics. We evaluated each individual potential confounder to identify those that altered the relevant prevalence ratio by > 5–10% irrespective of statistical significance [15]; these were further evaluated in multivariable log-binomial regression models. Percent reductions in HRHPV16 and 18 were quantified as (1–adjusted prevalence ratio) × 100.

Statistical analysis was performed using STATA version 15.1. To identify risk factors associated with HPV positivity, univariate and separate unconditional multivariate logistic regression models were used to compare demographic and disease-associated characteristics between participants who tested positive and participants who tested negative for HRHPV16, 18 and other tested HRHPV genotypes.

For the immunological analysis we compared the geometric mean titres (GMTs) of the HPV-specific NAb titres against HRHPV-16, and -18 in girls who previously received one dose of 4vHPV with girls who received no vaccine. NAbs titres were log-transformed and compared using the unpaired Student’s *t*-test. These analyses were performed using GraphPad Prism software, version 5.0. Our chosen sample size provided 90% power to detect a 30% difference in HPV antibodies with a 2-sided 5% significance level.

Knowledge-based questions comprised answers of ‘yes’, ‘no’, ‘don’t know’. Participant answers were transcribed in Microsoft Excel and were assigned low, moderate and high scores based on total outcome. Blank answers on an otherwise completed questionnaire were classified as incorrect. Attitudes-based questions allowed for multiple responses [13].

## 3 Results

### 3.1 Participant demographics

The study enrolled a total of 475 women aged 16–26 years; 118 one-dose recipients and 357 unvaccinated women. Participant demographics according to vaccination status are detailed in Table 1. The mean age at recruitment was 20.5 (*SD =* 1.5) and 20.3 (*SD =* 1.6) years for vaccinated and unvaccinated groups respectively. Around 60% of participants identified as students at the time of enrolment, with a larger proportion (p = 0.007) of students represented in the unvaccinated group (242, 68%) compared to vaccinated (62, 53%). A greater proportion (p = 0.004) of vaccinated participants (19, 16%) indicated a monthly average income in the highest bracket when compared to unvaccinated participants (20, 6%). The majority of participants (320, 67%) had either engaged in sexual intercourse (311, 65%) or genital skin contact with a partner (9, 2%). This was consistent across both groups whereby 82 (69%) vaccinated and 238 (67%) unvaccinated participants had engaged in either sexual intercourse or genital skin contact.

**Table 1:** Demographic characteristics of participants according to vaccination status (n = 475).

Characteristic	Variable	Vaccinated cohort (1-dose)	Unvaccinated cohort	
		n (%)	n (%)	p value
All participants (N)		118 (25)	357 (75)	
Age (years) at assessment	16–19	43 (36)	161 (45)	0.19
	20–21	50 (42)	140 (39)	
	22–26	25 (21)	56 (16)	
Mean age at recruitment (years)		20.5 (*SD =* 1.5)	20.3 (*SD =* 1.6)	0.23
Place of recruitment	City district	89 (75)	265 (74)	0.80
	Rural province	29 (25)	92 (26)	
Relationship status	Single	59 (50)	175 (49)	0.96
	Couple, not living together	27 (23)	74 (21)	
	Couple living together	14 (12)	47 (13)	
	Divorced/separated	3 (3)	13 (4)	
	Married	15 (13)	46 (13)	
	No answer	0 (0)	1 (<1)	
Highest level of education	Did not graduate from junior school	2 (2)	0 (0)	0.08
	Junior school	2 (2)	14 (4)	
	High school	60 (51)	163 (46)	
	College/university	53 (45)	176 (49)	
	No answer	1 (<1)	4 (1)	
Employment status	Employed^1^	39 (33)	66 (18)	0.007
	Unemployed^2^	17 (14)	48 (13)	
	Student	62 (53)	242 (68)	
	No answer	0 (0)	1 (<1)	
			
Income (monthly average after tax)	0₮-200000₮ (lowest livelihood level)	61 (52)	199 (56)	0.004
	200001₮–500000₮	18 (15)	60 (17)	
	500001₮ or more	19 (16)	20 (6)	
	No answer	20 (17)	78 (22)	
			
Religion	None	45 (38)	141 (39)	0.93
	Buddhism	48 (41)	142 (40)	
	Christianity	6 (5)	24 (7)	
	Muslim	1 (<1)	1 (<1)	
	Other	12 (10)	31 (9)	
	No answer	6 (5)	18 (5)	
			
Smoking status	Yes	17 (14)	42 (12)	0.45
Alcohol Consumption	Yes	73 (62)	225 (64)	0.82
Sexually active	Yes	80 (68)	231 (65)	0.54
Genital skin contact^3^	Yes	2/35 (6)	7/126 (6)	0.97

1. Employed includes employed, but away from work (e.g. holiday, paid/maternity or child care leave), and herders.

2. Unemployed includes unemployed and looking for work.

3. Only for non-sexually active participants.

### 3.2 HPV prevalence

**Fig. 1:**
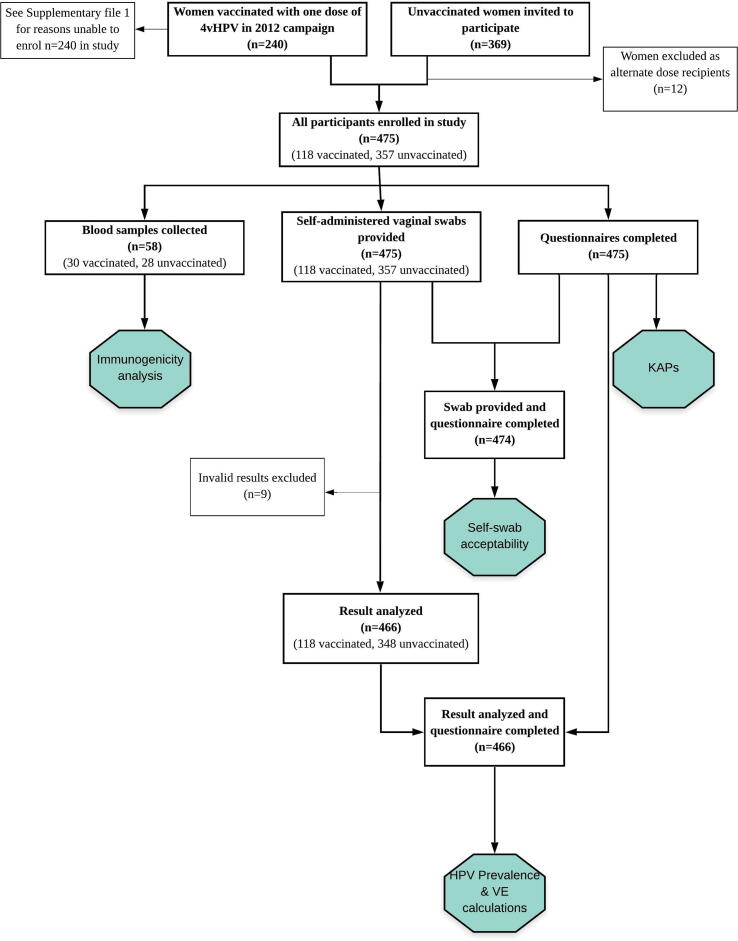
Flowchart of study design and results included for analysis. This figure depicts the study design, eligible participants and final participant numbers for each stage of the study.

Self-administered vaginal swabs were collected from all 475 women for HPV detection. From these, nine swabs returned invalid/error results and were excluded from analysis (Fig. 1). A resulting 466 swabs gave valid results; 118 from the vaccinated group and 348 from the unvaccinated group. HPV prevalence and prevalence ratios are presented in Table 2. Prevalence for all detected HPV genotypes was 27.1% (95%CI 19.3–36.1) and 35.3% (95%CI 30.3–40.6) for the vaccinated and unvaccinated groups, respectively. With vaccine-targeted HRHPV16 and 18 we noted a 92% reduction (95%CI 44–99) in detection among the vaccinated group [1.1% (95%CI 0.3–6.2)] compared with the unvaccinated group [15.4% (95%CI 11.3–20.3)]. In addition, one dose of 4vHPV led to a 90% reduction in HRHPV16 [aPR 0.10 (95%CI 0.01–0.73)] and an 89% reduction in HRHPV18 detection [aPR 0.11 (95%CI 0–0.70)] among vaccinated participants.

**Table 2:** HPV prevalence and prevalence ratios for vaccinated and unvaccinated participants.

Vaccinated cohort n/N	HPV prevalence in vaccinated participants (%) (95% CI)	Unvaccinated cohort n/N	HPV prevalence in unvaccinated participants (%) (95% CI)	Unadjusted prevalence ratio (95% CI)	Adjusted prevalence ratio (95% CI)
**Any HRHPV positive**					
32/118	27.1 (19.3–36.1)	123/348	35.3 (30.3–40.6)	0.77 (0.55–1.07)	0.67 (0.47–0.95)^a^
**HRHPV 16/18**					
1/87	1.1 (0.3–6.2)	41/266	15.4 (11.3–20.3)	0.07 (0.01–0.53)	0.08 (0.01–0.56)^a^
**Other high risk HPV types (excluding HPV 16/18)**					
31/117	26.5 (18.8–35.5)	82/307	26.7 (21.8–32.0)	0.99 (0.70–1.41)	0.83 (0.56–1.23)^a^
**HRHPV16**					
1/87	1.1 (0.03–6.2)	27/252	10.7 (7.2–15.2)	0.11 (0.01–0.78)	0.10 (0.01–0.73)^a^
**HRHPV18**					
0/86	0 (0–4.2)	16/241	6.6 (3.8–10.5)	0.12 (0–0.73)^b^	0.11 (0–0.70)^a,b^
**Multiple versus single genotypes^c^**					
0/32	0 (0–10.9)	27/123	21.9 (15.0–30.3)	0.10 (0–0.56) ^b^	0.11 (0–0.64)^a,b^

(a) Adjusted for employment status and income.

(b) Exact logistic regression.

(c) Single genotype = HRHPV16 only, HRHPV18 only or another HRHPV genotype only. Multiple genotypes = HRHPV16 + another HRHPV genotype or HRHPV18 + another HRHPV genotype or HRHPV16 + HRHPV18 + another HRHPV genotype.

Positivity for HRHPV16 and 18 was associated with having two to four lifetime sexual partners (aOR 3.01, 95%CI 1.32–6.83, p = 0.009; Table 3). It is important to note that no vaccinated participants tested positive for HRHPV18 and only one tested positive for HRHPV16 which could have been prevalent infection. The association of HPV positivity and lifetime sexual partners presented here is therefore based predominantly on unvaccinated participant data. Positivity for the other HRHPV types was associated with being in a current relationship (aOR 1.98, 95%CI 1.28–3.08, p = 0.002; Table 4).

**Table 3:** Factors associated with positivity of HRHPV16 and 18 type detection (n = 353).

Characteristic	HPV positive	HPV negative	Univariate analysis			Multivariate analysis
	n (%)	n (%)	OR (95% CI)	p value	aOR (95% CI)	p value
Age groups	N = 42	N = 311				
16–19 years	17 (40)	142 (46)	Ref			
20–21 years	17 (40)	123 (40)	1.15 (0.57–2.36)	0.69		
22–26 years	8 (20)	46 (15)	1.45 (0.59–3.59)	0.42		
Site	N = 42	N = 311				
City District	32 (76)	231 (74)	Ref			
Rural Province	10 (24)	80 (26)	0.90 (0.42–1.92)	0.79		
Relationship	N = 42	N = 310				
Not in current relationship	17 (40)	180 (58)	Ref			
In current relationship	25 (60)	130 (42)	2.04 (1.06–3.92)	0.03		
Education	N = 42	N = 308				
Primary	2 (5)	7 (2)	Ref			
Secondary	17 (40)	147 (48)	0.40 (0.08–2.11)	0.28		
Tertiary	23 (55)	154 (50)	0.52 (0.10–2.67)	0.44		
Employment	N = 42	N = 310				
Employed	9 (22)	61 (20)	Ref			
Unemployed	6 (14)	45 (14)	0.90 (0.30–2.72)	0.86		
Student	27 (64)	204 (66)	0.90 (0.40–2.01)	0.79		
Smokes tobacco	N = 42	N = 311				
Yes	8 (19)	27 (9)	2.47 (1.04–5.88)	0.04		
Consumes alcohol	N = 42	N = 309				
Yes	29 (69)	187 (61)	1.46 (0.73–2.91)	0.29		
Sexually active	N = 42	N = 311				
Yes	34 (81)	168 (54)	3.62 (1.62–8.07)	0.002		
Age at sexual debut	N = 34	N = 167				
8–15 years	3 (9)	3 (2)	Ref			
16–18 years	11 (32)	87 (52)	0.13 (0.02–0.70)	0.02		
>18 years	20 (59)	77 (46)	0.26 (0.05–1.39)	0.12		
Age of first partner	N = 33	N = 166				
<18 years	2 (6)	14 (8)	Ref			
>=18 years	31 (94)	152 (92)	1.43 (0.31–6.60)	0.65		
Age difference with first partner	N = 33	N = 165				
Partner younger	4 (12)	12 (7)	Ref			
0–3 years older	21 (64)	121 (73)	0.52 (0.15–1.77)	0.29		
3–11 years older	8 (24)	32 (20)	0.75 (0.19–2.96)	0.68		
Number of sexual partners in last 12 months	N = 31	N = 154				
1	26 (84)	140 (91)	Ref			
2	4 (13)	12 (8)	1.79 (0.54–6.00)	0.34		
3–6	1 (3)	2 (1)	2.69 (0.24–30.79)	0.43		
Number of lifetime sexual partners	N = 34	N = 163				
1	17 (50)	113 (69)	Ref			
2–4	15 (44)	45 (28)	2.21 (1.02–4.81)	0.04	3.01 (1.32–6.83)	0.009
≥5	2 (6)	5 (3)	2.66 (0.48–14.81)	0.26	3.25 (0.52–20.26)	0.21
Prior pregnancy	N = 34	N = 165				
Yes	17 (50)	90 (54)	0.83 (0.40–1.74)	0.63		
Genital skin contact	N = 37	N = 279				
Yes	10 (27)	31 (11)	2.96 (1.31–6.70)	0.009		
Oral sex	N = 42	N = 305				
Yes	5 (12)	29 (10)	1.29 (0.47–3.53)	0.62		
Received 4vHPV	N = 42	N = 311				
Yes	1 (2)	86 (28)	0.06 (0.01–0.47)	0.007	0.05 (0.01–0.42)	0.005

**Table 4:** Factors associated with positivity of other high-risk HPV type detection (excluding HRHPV16 and 18) n = 424.

Characteristic	HPV positive	HPV negative	Univariate analysis			Multivariate analysis
	n (%)	n (%)	OR (95% CI)	p value	aOR (95% CI)	P value
Age groups	N = 112	N = 311				
16–19 years	41 (37)	142 (46)	Ref			
20–21 years	45 (40)	123 (39)	1.26 (0.78–2.06)	0.34		
22–26 years	26 (23)	46 (15)	1.96 (1.08–3.54)	0.03		
Site	N = 113	N = 311				
City District	83 (73)	231 (74)	Ref			
Rural Province	30 (27)	80 (26)	1.04 (0.64–1.70)	0.86		
Relationship	N = 112	N = 310				
Not in current relationship	46 (41)	180 (58)	Ref			Ref
In current relationship	66 (59)	130 (42)	1.99 (1.28–3.08)	0.002	1.99 (1.28–3.08)	
Education	N = 111	N = 308				
Primary	9 (8)	7 (2)	Ref			
Secondary	55 (50)	147 (48)	0.29 (0.10–0.82)	0.02		
Tertiary	47 (42)	154 (50)	0.24 (0.08–0.67)	0.007		
Employment	N = 113	N = 310				
Employed	32 (28)	61 (20)	Ref			
Unemployed	14 (12)	45 (14)	0.59 (0.28–1.24)	0.17		
Student	67 (60)	204 (66)	0.63 (0.38–1.04)	0.07		
Smokes tobacco	N = 113	N = 311				
Yes	21 (19)	27 (9)	2.40 (1.29–4.45)	0.005		
Consumes alcohol	N = 111	N = 309				
Yes	77 (69)	187 (61)	1.48 (0.93–2.35)	0.10		
Sexually active	N = 113	N = 311				
Yes	105 (93)	168 (54)	11.17 (5.26–23.71)	<0.001		
Age at sexual debut	N = 104	N = 167				
8–15 years	2 (2)	3 (2)	Ref			
16–18 years	60 (58)	87 (52)	1.03 (0.17–6.38)	0.97		
>18 years	42 (40)	77 (46)	0.82 (0.13–5.09)	0.83		
Age of first partner	N = 101	N = 166				
<18 years	9 (9)	14 (8)	Ref			
>=18 years	92 (91)	152 (92)	0.94 (0.39–2.26)	0.89		
Age difference with first partner	N = 101	N = 165				
Partner younger	9 (9)	12 (7)	Ref			
0–3 years older	63 (62)	121 (73)	0.69 (0.28–1.73)	0.44		
3–11 years older	29 (29)	32 (20)	1.21 (0.44–3.28)	0.71		
Number of sexual partners in last 12 months	N = 95	N = 154				
1	75 (79)	140 (91)	Ref			
2	15 (16)	12 (8)	2.33 (1.04–5.24)	0.04		
3–6	5 (5)	2 (1)	4.67 (0.88–24.63)	0.07		
Number of lifetime sexual partners	N = 100	N = 163				
1–2	76 (76)	141 (86)	Ref			
≥3	24 (24)	22 (14)	2.02 (1.06–3.85)	0.03		
Prior pregnancy	N = 102	N = 165				
Yes	46 (45)	90 (54)	0.68 (0.42–1.12)	0.13		
Genital skin contact	N = 91	N = 279				
Yes	13 (14)	31 (11)	1.33 (0.66–2.67)	0.42		
Oral sex	N = 109	N = 305				
Yes	15 (14)	29 (10)	1.52 (0.78–2.96)	0.22		
Received 4vHPV	N = 113	N = 311				
Yes	31 (27)	86 (28)	0.99 (0.61–1.60)	0.96	0.98 (0.60–1.60)	0.95

### 3.3 HPV immunogenicity

**Fig. 2:**
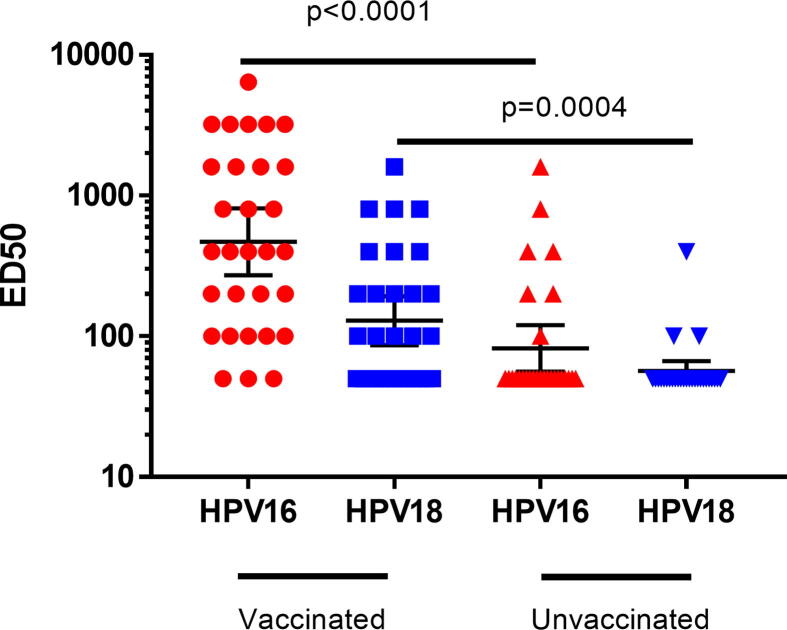
Neutralising antibody responses to HRHPV16 and 18, six years following one dose of 4vHPV. Data is presented as GMT ± 95%CI. ED50: effective dose 50. An HPV pseudovirion-based neutralisation assay was used to detect neutralising antibodies (NAb) to HRHPV16 and 18 [10]. The effective dose 50 (ED50) is the highest serum dilution that reduces the secreted alkaline phosphatase activity by at least 50% in comparison to the control (pseudovirions without serum). A sample with an ED50 value of ≥ 100 was considered HPV seropositive; seronegative samples were given a value of 50.

Of the 30 blood samples from vaccinated participants, 90% (27) remained seropositive for HRHPV16 and 58% (17) remained seropositive for HRHPV18 after six years. None of the seropositive, vaccinated women had HRHPV16 or 18 detected in vaginal swabs, however eight tested positive for other HRHPV genotypes. Among the 28 blood samples from unvaccinated women, 25% (7) were seropositive to HRHPV16 and 10% (3) seropositive to HRHPV18; all ten were negative for current HRHPV16 or 18 infection. Significantly higher geometric mean NAb titers to HRHPV16 and 18 were observed in one-dose vaccinated women compared with unvaccinated women (HRHPV16: 470.2 (95%CI: 272.2–812.3) vs. 82.0 (95%CI: 56.2–119.8); HRHPV18: 128.9 (95%CI: 86.5–192.2) vs. 56.6 (95%CI: 48.0–66.7)) (Fig. 2).

### 3.4 Questionnaire results

The knowledge and attitudes questionnaire results overall were consistent with our previous study [13], so only a brief overview will be provided. All 475 participants completed the knowledge and attitudes questionnaire. There was an overall low level of knowledge on HPV, HPV vaccines or cervical cancer. From the seven questions related to HPV, no participants answered all questions correctly. One participant correctly answered six of the seven questions, however, the majority of women (86%) did not achieve 50% or greater correct. From the three questions related to HPV vaccines, 37 participants (7.8%) answered all questions correctly. The majority of participants (70%) did not answer more than one question correctly. Regarding cervical cancer, 392 participants (82.5%) had not heard of cervical screening or the Papanicolaou smear (cervical smear) test. These results were consistent across both vaccinated and unvaccinated groups (data not shown).

A total of 474 participants answered all questions regarding self-swab acceptability. Almost all participants (469, 98.9%) identified that the self-swab instructions were clear and exhaustive, 389 participants (82.0%) indicated that the technique was easy to conduct and 317 (66.9%) reported they did not experience any pain during the process. A greater number of participants (284, 59.9%) identified that they would prefer to perform the swab in an outpatient clinic rather than at home.

## 4 Discussion

This study builds on previous follow-up studies of HPV demonstration campaigns [7], [8] by measuring the impact of a single dose of 4vHPV six years following a 2012 pilot vaccination campaign in Mongolia. The results highlight that a single dose of 4vHPV substantially reduced vaccine-type HRHPV 16/18 detection rates and elicited sustained seropositivity for vaccine-targeted HPV types among vaccinated women. This study demonstrated a 90% reduction of HRHPV16 and 89% reduction of HRHPV18 detection among participants vaccinated with one dose of 4vHPV six years prior when compared to unvaccinated participants. The results of this study are strengthened by the similar prevalence of non-vaccine HRHPV types between both study groups (26.5% and 26.7%) which indicates comparable exposure. The overall prevalence of any type of HPV tested among all study participants was 31.2%. This is slightly lower than the 36% prevalence found in a study among 110 women attending a sexually transmitted infection (STI) clinic in Ulaanbaatar [16] and the 39.5% prevalence found in our earlier study of three-dose 4vHPV recipients [13]. However, our previous study had a higher percentage (72.8%) of participants who were sexually active [13]. This high HPV rate among young Mongolian women contributes to an emerging body of data that justifies implementation of a national HPV vaccine program in Mongolia. Positivity for HRHPV16 and 18 was associated with having two to four lifetime sexual partners, while positivity for the other HRHPV types was associated with being in a current relationship. These findings are consistent with other studies exploring HPV-associated risk factors [17], [18], [19].

The immunogenicity results extend current evidence of persisting immune responses following a single dose of 2vHPV or 4vHPV [4], [5], [6], [7], [8]. The lower seropositive rates for HRHPV16 (90%) and HRHPV18 (58%) in our study compared to studies from India (97% and 67.2%, respectively) and Fiji (95% and 68%, respectively) is likely due to the small sample size as measured by the HPV pseudovirion neutralisation assay. A lower seropositivity rate for HRHPV18 compared with HRHPV16 may suggest a more rapidly waning immune response and argues for continued surveillance for any vaccine failures. It is possible that the antigenicity of HRHPV18-based antigens used in the assay is lower than HRHPV16-based antigens, although lower immunogenicity of HRHPV18 vaccine responses have been documented previously [20], [21]. Lower HRHPV18 NAb may also be partly due to lower circulating HRHPV18 in the community [13] and therefore less immunological boosting. There is currently no identified level of NAb that has been shown to protect against HPV infection and so the clinical relevance is unclear. Previous or current infections may also have influenced NAb responses, but these are unlikely to explain the high seropositivity rates and NAb titres in vaccinated women compared with unvaccinated women.

The questionnaire results were consistent with our larger study with three-dose recipients [13], demonstrating a low level of knowledge among young Mongolian women in relation to HPV, the vaccine and cervical cancer. In this current study, a higher proportion of participants were university educated (47% vs. 18.8% in our earlier study), strengthening the need for better education and awareness in Mongolia in relation to HPV and cervical cancer. The questionnaire results also demonstrated that the self-swab technique was acceptable and easy to perform. This is important for future cervical screening considerations in Mongolia and potentially other similar LMIC settings.

A limitation of this study was the differential testing methods between the Cepheid and Seegene technology. The original method was based on limited cost-effective technology availability in Mongolia and additional funds were sourced to retest the HRHPV18/45 positive samples only. Additional limitations include the limited sample size of vaccinated women and the fact that girls were self-chosen (or parent-chosen) to receive only one dose of vaccine and we therefore could not account for potential selection bias. However, the sample size was sufficiently powered to demonstrate a difference in HPV detection between the groups. A strength of this study was the consistency in the HPV prevalence and immunogenicity data with the Fiji and Uganda studies [7], [8], although there were no data from the two-or three-dose schedules to evaluate the difference in antibody magnitude. The greatest strength of this study is that it was performed six years after vaccination, with paired prevalence and immunogenicity data, representing a valuable contribution to the data on sustained protection against vaccine-targeted HPV following one dose of 4vHPV.

Since WHO revised the schedule recommendations for 2vHPV and 4vHPV from three to two doses separated by six months for girls under 15 years [3], there has been growing global interest in the potential protection provided by a single dose of HPV vaccine. Following targeted-actions by key stakeholders, including presentation of results from our three-dose study [13], the Mongolian Ministry of Health has approved phased introduction of the HPV vaccine in-country from 2020 to 2022. Specific implementation protocols and scheduling are still under discussion and the results of this study provide an important resource for context-specific decisions. There are ongoing randomised controlled trials from Tanzania, (DoRIS), Costa Rica (PRIMAVERA-ESCUDDO) and Kenya (KEN SHE) [22], [23], [24], [25] as well as a community intervention effectiveness study in Thailand (IVIHPV1) [26] and an observer-blind non-inferiority trial with 9vHPV in The Gambia (HANDS) [27] examining non-inferiority of one-dose HPV vaccination schedules. As we await the results of these trials, our study provides a suggestion of long-term protection (at least six years) offered by a single dose of HPV vaccine, which is in keeping with other observational data. The current global supply of HPV vaccines is not sufficient to meet the projected demand until 2024 [28]. In light of the global vaccine shortage, and especially for LMICs obstructed by the financial commitment of vaccine introduction, these results offer a potential solution for HPV vaccine introduction.

## 5 Conclusion

This study demonstrates that one dose of 4vHPV, administered six years prior, is associated with reduced HPV detection rates and sustained vaccine-type HPV seropositivity among young Mongolian women aged 16–26 years. These results contribute to the emerging global evidence regarding single-dose HPV vaccine schedules. These results are pertinent whilst we await the results of RCTs on one dose in an era of vaccine shortage. The study results also contribute to the limited HPV prevalence data available in Mongolia and demonstrate an acceptance of HPV self-testing among young Mongolian women. The authors foresee the results of this study contributing to greater efforts to reduce the barriers for HPV vaccine uptake in LMICs.

## 6 Ethical considerations

This study was approved by the Ethical Committee of the Mongolian Ministry of Health (#70, 24th August 2018), the Human Research Ethics Committee (HREC) (Reference Number: HREC/18/RCHM/132) and the Royal Children’s Hospital, Melbourne Human Research Ethics Committee (RCH HREC Reference Number: 38046A).

## Funding

This study was funded by the 10.13039/100000865Bill & Melinda Gates Foundation (OPP118417). The study sponsor was not involved in any stage of the study design, data collection, analysis or publication process. The corresponding author confirms that she had full access to all study data and had final responsibility for the decision to submit for publication.

## Authors’ contributions

CvM, FMR, TB and KM designed the study. KM, CvM, FMR, SMG and US provided scientific input. TB, MM, TK and MTD collected or generated study data. CvM, ZQT and PL conducted data analysis and provided technical statistical input. ZQT, PL and NN provided technical laboratory support. CvM, ZQT, PL and MTD interpreted the data. MTD prepared Fig. 1, ZQT prepared Fig. 2. MTD drafted the first edition of the paper. All authors reviewed and approved the final version for submission. All authors attest they meet the ICMJE criteria for authorship.

## Declaration of Competing Interest

The authors declare the following financial interests/personal relationships which may be considered as potential competing interests: Professor Suzanne Garland (SMG) is a member of 9vHPV Advisory Board, Merck Scientific and Global Advisory Boards. SMG has received Grants through her institution from Merck for HPV studies in young women. All other authors have no conflicts of interest to declare.
