# Supplementary material for: A single dose of quadrivalent human papillomavirus (HPV) vaccine is immunogenic and reduces HPV detection rates in young women in Mongolia, six years after vaccination
Source: Vaccine. 2020 Jun 2;38(27):4316–24. doi: 10.1016/j.vaccine.2020.04.041 (PMC7254061; doi:10.1016/j.vaccine.2020.04.041)
Supplement: Supplementary data 1 [file mmc1.docx]

**Supplementary file 1. Description of laboratory methods used based on previous study of three dose 4vHPV recipients in Mongolia (referenced in paper).**

- If participant consented to self-collect vaginal swab, research assistants provided participants with detailed pictorial instructions and a verbal description on recommended collection techniques.
- All samples were stored and labeled according to the unique participant ID.
- The swabs were stored in Preserv Cyt solution (ThinPrep Pap Test, Hologic Inc, Malborough, MA, USA) at room temperature.
- At Onoshmed laboratory in Ulaanbaatar, one mL of each Preserv Cyt sample was tested by Xpert HPV Assay (Cepheid Inc, Sunnyvale, CA, USA).
- Xpert HPV Assay: a real-time PCR system with integrated automated sample processing, cell lysis, purification, nucleic acid amplification and qualitative E6/E7 region detection of the viral DNA genome of HRHPV types.
- The system identifies HRHPV16 and HRHPV18/45 types in two distinct detection channels, and reports 11 other high risk types (P3 channel: 31, 33, 35, 52, 58; P4 channel: 51, 59; P5 channel: 39, 56, 66 and 68) in a pooled result.
- Samples testing 18/45 positive (n=30) were retested using the Anyplex II HPV 28 (Seegene, Korea) which can distinguish 28 HPV genotypes (6, 11, 16, 18, 26, 31, 33, 35, 39, 40, 42, 43, 44, 45, 51, 52, 53, 54, 56, 58, 59, 61, 66, 68, 69, 70, 73, 82).
- Where results differed, the Seegene results were used for final analysis.
