# Supplementary material for: A single dose of quadrivalent human papillomavirus (HPV) vaccine is immunogenic and reduces HPV detection rates in young women in Mongolia, six years after vaccination
Source: Vaccine. 2020 Jun 2;38(27):4316–24. doi: 10.1016/j.vaccine.2020.04.041 (PMC7254061; doi:10.1016/j.vaccine.2020.04.041)
Supplement: Supplementary data 2 [file mmc2.docx]

**Supplementary file 2. Recruitment efforts for one-dose 4vHPV recipients**

Table A). Reasons for vaccinated women not enrolling in study

| **Study Site** | **Unable to locate^+^** | **Did not consent to participate** | **Studying/living abroad** | **Incorrect ID in initial NCCD database** | **Died** | **Moved to remote/ unknown location** | **Recently delivered/ currently pregnant** | **Enrolled in study** | **TOTAL** |
| --- | --- | --- | --- | --- | --- | --- | --- | --- | --- |
| **Baganuur district** | 1 | - | - | - | - | - | 1 | 5 | 7 |
| **Bayangol district** | 32 | 17 | 29 | 9 | 4 | 5 | 3 | 84 | 183 |
| **Selenge province** | 2 | 1 | - | 1 | - | - | - | 2 | 6 |
| **Umnugobi province** | 4 | 1 | 1 | - | - | 4 |  | 27 | 37 |
| **TOTAL** | 39 | 19 | 30 | 10 | 4 | 9 | 4 | 118 | 233 |

Table B). Average number of phone calls made to vaccinated women for invitation to study

| **Enrolment status/reason for not enrolling** | **Total number of calls made** | **Average number of calls made per participant** |
| --- | --- | --- |
| **Unable to locate^+^** | 260 | 6.7 |
| **Did not consent** | 64 | 3.4 |
| **Studying/living abroad** | 80 | 2.7 |
| **Incorrect ID in initial database** | 20 | 2 |
| **Died** | 6 | 1.5 |
| **Moved to remote/unknown location** | 19 | 2.1 |
| **Recently delivered/currently pregnant** | 8 | 2 |
| **Enrolled participants** | 536 | 4.5 |

^+^Unable to locate meant we could not find current contact information for the women. This included seeking assistance from health departments and telecommunications companies.
